# Early Resuscitation in Paediatric Sepsis Using Inotropes – A Randomised Controlled Pilot Study in the Emergency Department (RESPOND ED): Study Protocol and Analysis Plan

**DOI:** 10.3389/fped.2021.663028

**Published:** 2021-05-31

**Authors:** Amanda Harley, Shane George, Megan King, Natalie Phillips, Gerben Keijzers, Debbie Long, Kristen Gibbons, Rinaldo Bellomo, Luregn J. Schlapbach

**Affiliations:** ^1^Child Health Research Centre, The University of Queensland, and Paediatric Intensive Care Unit, Queensland Children's Hospital, Brisbane, QLD, Australia; ^2^School of Nursing, Midwifery and Social Work, University of Queensland, Brisbane, QLD, Australia; ^3^Department of Emergency Medicine, Gold Coast University Hospital, Southport, QLD, Australia; ^4^Emergency Department Queensland Children's Hospital, Brisbane, QLD, Australia; ^5^School of Medicine, Griffith University, Southport, QLD, Australia; ^6^Faculty of Health Sciences and Medicine, Bond University, Southport, QLD, Australia; ^7^School of Nursing, Centre of Healthcare Transformation, Queensland University of Technology, Brisbane, QLD, Australia; ^8^Department of Intensive Care, Austin Health, Heidelberg, VIC, Australia; ^9^Pediatric and Neonatal Intensive Care Unit, Children's Research Center, University Children's Hospital Zurich, Zurich, Switzerland

**Keywords:** sepsis, septic shock (MeSH), pediatric, fluid, resuscitation, inotrope, trial

## Abstract

**Introduction:** Septic shock in children still carries substantial mortality and morbidity. While resuscitation with 40–60 mL/kg intravenous fluid boluses remains a cornerstone of initial resuscitation, an increasing body of evidence indicates potential for harm related to high volume fluid administration. We hypothesize that a protocol on early use of inotropes in children with septic shock is feasible and will lead to less fluid bolus use compared to standard fluid resuscitation. Here, we describe the protocol of the Early Resuscitation in Paediatric Sepsis Using Inotropes – A Randomised Controlled Pilot Study in the Emergency Department (RESPOND ED).

**Methods and analysis:** The RESPOND ED study is an open label randomised controlled, two arm, multicentre pilot study conducted at four specialised paediatric Emergency Departments. Forty children aged between 28 days and 18 years treated for presumed septic shock will be randomized in a 1:1 ratio to early inotropes vs. standard fluid resuscitation. Early inotrope treatment is defined as the commencement of a continuous intravenous adrenaline infusion after 20 mL/kg fluid bolus resuscitation. Standard fluid resuscitation is defined as delivery of 40 to 60 mL/kg fluid bolus resuscitation prior to commencement of inotropes. In addition to feasibility outcomes, survival free of organ dysfunction censored at 28 days will be assessed as the main clinical outcome. The study cohort will be followed up at 28 days, and at 6 months post enrolment to assess quality of life and functional status. Biobanking nested in the study cohort will be performed to enable ancillary biomarker studies.

**Ethics and dissemination:** The trial has ethical clearance (Children's Health Queensland, Brisbane, HREC/18/QCHQ/49168) and is registered in the Australian New Zealand Clinical Trials Registry (ACTRN12619000828123). Enrolment commenced on July 21st, 2019. The primary manuscript will be submitted for publication in a peer-reviewed journal.

**Trial Registration:** Australian and New Zealand Clinical Trials Registry, ACTRN12619000828123.

## Introduction

Sepsis, characterised by infection with associated organ dysfunction (1, 2), represents one of the leading acutely life-threatening conditions in pediatric Emergency Departments (ED) (3). The burden of sepsis on child health extends beyond mortality (4, 5) to morbidity and long-term sequelae which may dramatically affect Quality of Life (QoL) after discharge (6, 7). Improving recognition and treatment of sepsis is a priority for the World Health Organisation (8), and regional initiatives to improve the treatment of children with sepsis are underway in several countries (9, 10). Currently, best practice comprises timely delivery of a bundle consisting of intravenous antibiotics, blood culture and lactate sampling, and intravenous fluid boluses (11). Improved risk-adjusted survival rates have been shown for children treated with a sepsis bundle within 1 h of sepsis recognition (12).

Recent recommendations for treatment of sepsis and septic shock in children include the American College of Critical Care Medicine clinical practice parameters (13), and the 2020 paediatric Surviving Sepsis Campaign (SSC) guidelines (11). While both advocate for the administration of 40–60 mL/kg of fluid bolus therapy prior to the commencement of intravenous inotropes, the 2020 SSC added caution for fluid bolus therapy if intensive care resources are unavailable given the evidence towards worse survival in children with severe infections observed in the Fluid Expansion as Supportive Therapy (FEAST) study (14). The aim of administering fluid boluses in paediatric septic shock is to restore haemodynamic function and support the cardiovascular system, ensuring adequate perfusion to organs via clinical endpoints such as normalisation of heart rate, blood pressure and capillary refill. An increasing body of observational studies suggests potential harm related to excessive fluid delivery, including prolonged ventilation, increased mortality and ICU length of stay (15). Emergency and intensive care physicians report ongoing high fluid use in sepsis, and a high variability exists in local practices (16, 17). The premise for use of intravenous inotropes stems from observational data indicating that children with septic shock frequently manifest myocardial dysfunction coupled with variable degrees of vasoplegia which may benefit from catecholamines such as adrenaline (18). To date, there are limited high-quality randomised-controlled trials (RCTs) investigating optimal resuscitation therapies for children with septic shock. The timing and amount of fluids administered remain one of the most controversial topics in sepsis resuscitation (19).

For this purpose, we designed the Early Resuscitation in Paediatric Sepsis Using Inotropes – A Randomised Controlled Pilot Study in the Emergency Department (RESPOND ED). This pragmatic pilot study tests the feasibility of a paediatric parallel group RCT comparing early inotropes vs. standard fluid resuscitation in children aged between 28 days and 18 years presenting with suspected septic shock. We hypothesized that a protocolised early commencement of intravenous inotropes is feasible and that early inotropes will reduce the volume of resuscitation fluids administered compared to standard sepsis resuscitation. Here, we describe the RESPOND ED study protocol and statistical analysis plan.

## Methods

### Study Design and Setting

**Figure 1:**
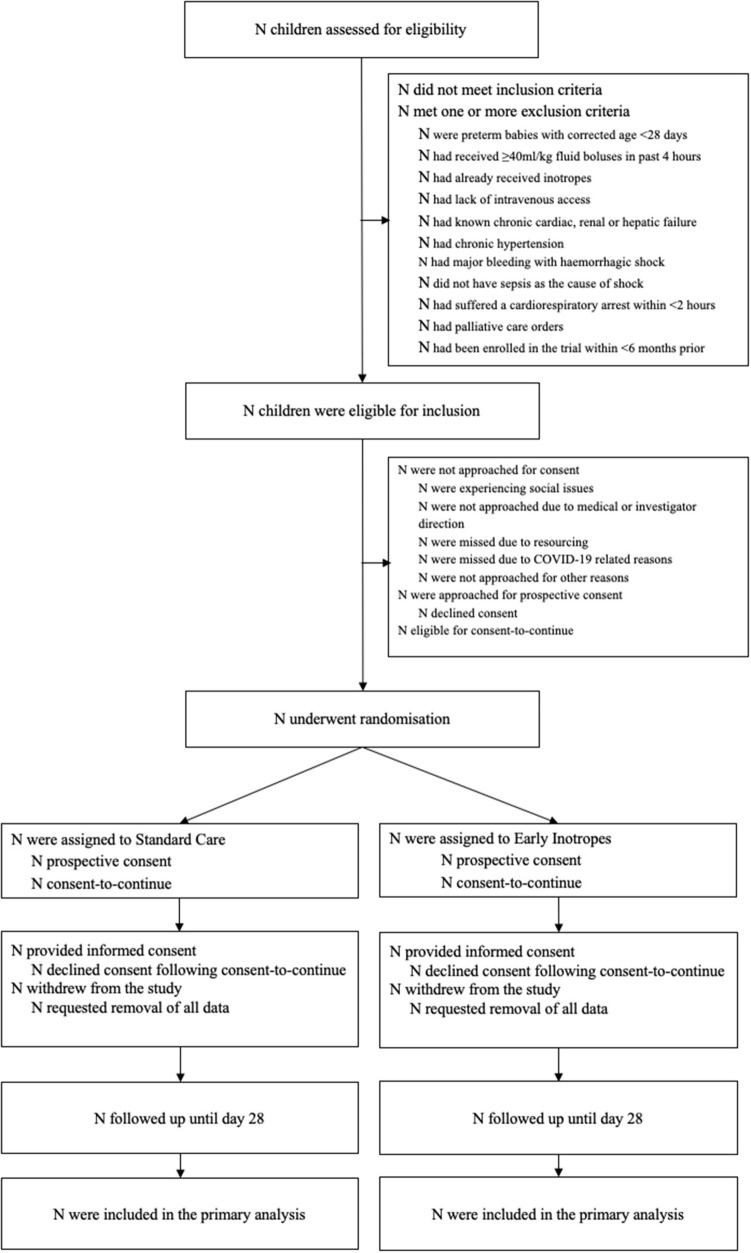
CONSORT participant flow diagram for RESPOND ED.

The RESPOND ED study is a pilot multicentre, open label, pragmatic randomized controlled trial (RCT) for children aged between 28 days and 18 years treated for septic shock (Figure 1). The study will recruit across four tertiary Emergency Departments (ED) and Paediatric Intensive Care Units (PICU) of participating sites in Queensland, Australia. The trial compares***early inotropes*** defined as adrenaline infusion started after 20 mL/kg fluid resuscitation, with ***standard care*** defined as providing up to 40–60 mL/kg fluid resuscitation prior to initiation of inotropes (11, 13). The study protocol has been approved by the Children's Health Queensland Hospital and Health Service Human Research Ethics Committee (HREC/18/QCHQ/49168) and registered with the Australian New Zealand Clinical Trials Registry (ACTRN12619000828123, June 2019). The trial abides by the Standard Protocol Items: Recommendations for Interventional Trials (SPIRIT) guidelines (20).

### Participants

#### Inclusion and Exclusion Criteria

Included will be children aged between 28 days and 18 years, where the treating clinician decides to treat for septic shock and administer intravenous antibiotics and an intravenous fluid bolus. Randomisation will occur after participants have received at least 20mL/kg fluid bolus in the last 4 h (or 1,000 mL fluid bolus in patients weighing over 50 kg), and the treating clinician must have decided to continue treating due to ongoing signs of shock. The exclusion criteria relate to patient characteristics which interfere with the study intervention and presence of chronic conditions which can interfere with the clinical outcome, as detailed in Table 1.

**Table 1:** Inclusion and exclusion criteria.

**Rule**	**Criterium**	**Definition**
Inclusion	Age	• Aged ≥28 days and <18 years
	Illness	• Treated for sepsis
	Treatment	• Received at least 20 ml/kg fluid bolus in the last 4 h and clinician decides to continue treating signs of shock
	Consent	• Parental/caregiver consent prior to or after enrolment
Exclusion	Age	• Preterm babies born <34 weeks gestation that have a corrected age of <28 days
	Treatment	• Received ≥40 mL/kg of fluid boluses during the 4 h pre-enrolment • Inotrope infusion commenced pre-enrolment • Lack of access (intraosseous, central venous or peripheral) to administer fluids and/or inotropes after 60 min of enrolment
	Co-morbidities	• Cardiomyopathy or chronic cardiac failure • Chronic hypertension due to cardiovascular or renal disease, requiring regular antihypertensive treatment. • Known chronic renal failure (defined as requiring renal replacement therapy) • Known chronic hepatic failure • Palliative care patient/patient with limitation of treatment (not for inotropes, cardiopulmonary resuscitation, extracorporeal membrane oxygenation, intubation or ventilation)
	Illness severity	• Cardiopulmonary arrest in the past 2 h requiring cardiopulmonary resuscitation of >2 min duration, or death is deemed to be imminent or inevitable during this admission. • Major bleeding with haemorrhagic shock • Sepsis is not likely to be the cause of shock
	Previous study enrolment	• Enrolment in RESPOND study <6 months prior

#### Screening and Consenting

Participants will predominantly be screened and recruited in the ED but can be recruited in PICU (Figure 1). Consent will be obtained from the guardians with the additional option to consent for biobanking. Where possible, prospective consent will be sought. Due to the emergency nature of septic shock, it is anticipated that in certain situations timely informed consent may not be feasible. In this circumstance a consent-to-continue approach will be applied [i.e., patients can be enrolled in the study if prospective consent cannot be obtained in a timely manner, but data can only be used in the analysis if the parents thereafter provide their written consent to continue in the study; also termed “deferred” or “delayed” consent (21)]. Informed consent will be sought from the parent/guardian by the study team or medical officer as soon as practical.

Screening will start at the time when the medical team decides to treat a patient for sepsis as per the institutional sepsis pathway (Queensland Paediatric Statewide Sepsis Pathway, which is being used at all study sites) to enable a pragmatic screening entry point. The trial does not mandate specific physiological thresholds to assess shock, rather the point of enrolment reflects the decision by the treating clinician to start a sepsis treatment bundle. Clinicians are advised to give a first fluid bolus of 20 mL/kg (or 1,000 mL fluid bolus in patients >50 kg) as per SSC guidelines irrespective of any decisions regarding enrolment in the study. Clinicians can give this amount of fluid as one bolus or split into aliquots of smaller amounts of bolus fluid.

### Randomisation

Patients will be allocated in a 1:1 ratio to the treatment group and standard care group (receiving early inotropes vs. standard fluid management). A permuted block randomisation method with variable block sizes of two, four and six and stratified by site will be used to allocate eligible patients to a study group. Randomisation will be performed by the site investigator, research coordinator or treating clinician by opening the next sequential sealed opaque envelope which are prepared using a randomisation sequence prepared by The University of Queensland, Brisbane, Australia.

### Blinding

No blinding will be performed; the intervention will be open labelled. The main aim of this pilot study is to ascertain the feasibility of the study protocol. Blinding of fluid vs. inotropes would be logistically almost impossible to achieve. In addition, given the study is enrolling acutely ill children on a trajectory of potential rapid deterioration, clinicians need to be able to access the amount of delivered interventions for safety reasons.

### Study Interventions

**Figure 2:**
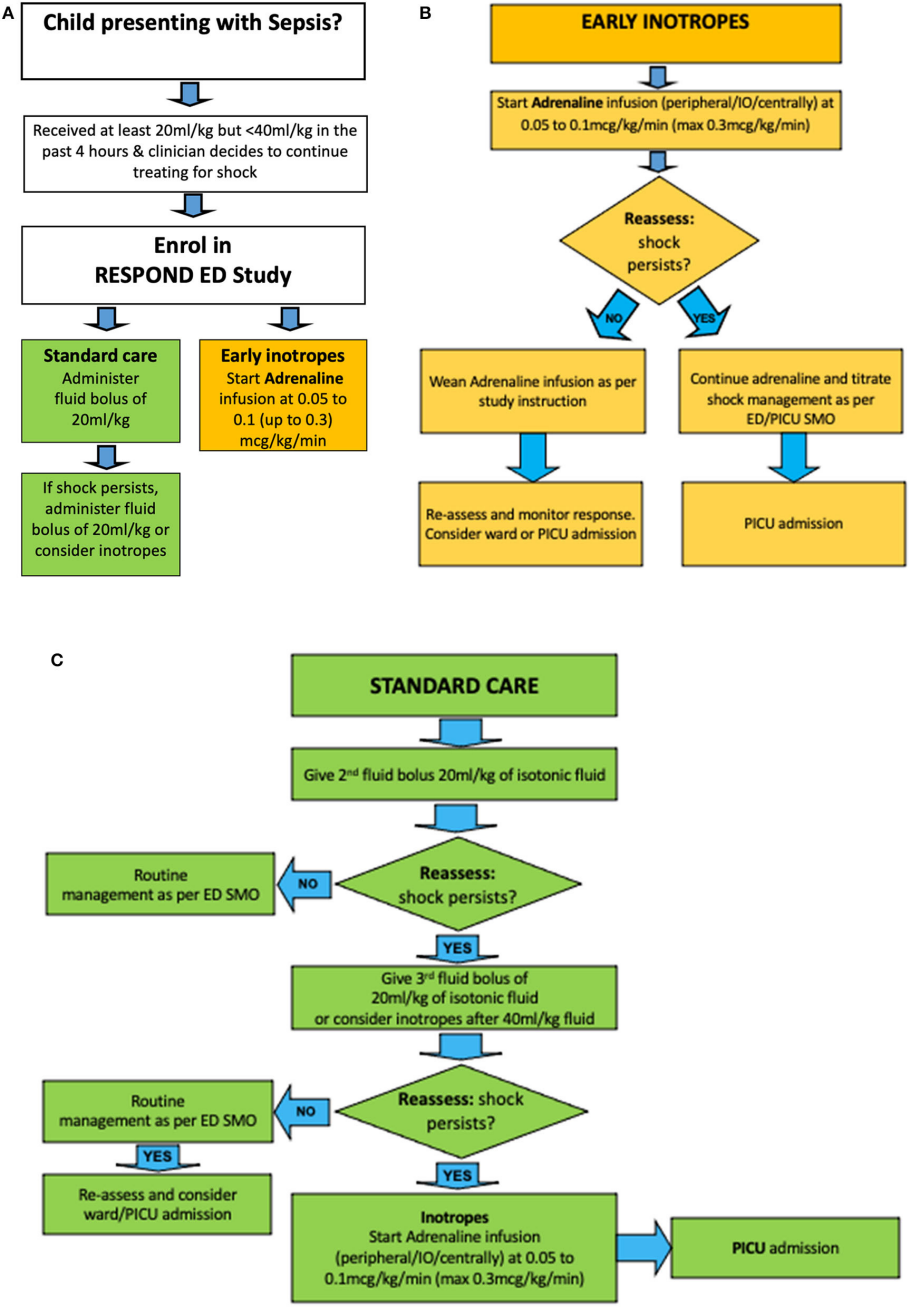
Treatment algorithm for RESPOND ED. Children with sepsis having received an initial fluid bolus are randomized **(A)** to *early inotropes*, defined as initiation of adrenaline infusion after 20 ml/kg fluid bolus **(B)**, vs. *standard care*, defined as provision of 40–60 ml/kg fluid boluses before inotropes are started **(C)**. ED SMO, Emergency Department Senior Medical Officer.

Allocated treatment will be started as soon as possible after randomisation. The allocated intervention arm will be administered if the clinician is continuing to treat for signs of septic shock. The trial compares ***early inotropes*** started after 20 mL/kg fluid resuscitation with ***standard care*** (Figure 2).

#### Early Inotropes

Patients in the intervention arm will receive an infusion of intravenous adrenaline immediately after randomisation, i. e. after the initial fluid bolus of 20 mL/kg (1,000 mL fluid bolus in patients >50 kg). Adrenaline will be initiated at 0.05 to 0.1 (up to 0.3) microgram/kg/min as per institutional guidelines. For the administration, adrenaline 1 mg will be diluted in 49 mls of 5% Dextrose. The dilution is used to hasten drug delivery and response to drug rate changes, to reduce risks with extravasation, and to facilitate peripheral application. Drug delivery will occur through dose error reduction software for infusion devices to ensure safe delivery of applied standardised drug concentrations. In emergency situations, adrenaline can be given through a peripheral intravenous, intraosseous, or central venous access line. Once started, adrenaline should be delivered for 60 min at a dose titrated to age-based physiological targets in patients who show signs of stability before weaning the drug. Patients who are successfully weaned off adrenaline within <4 h can in principle be admitted to the ward, yet should be reviewed by PICU staff within 4–6 h after transfer from ED. In patients deteriorating despite adrenaline infusion, the dose rate should be increased and admission to PICU with appropriate monitoring sought as per local practice.

#### Standard Care

In the standard arm, patients will receive standard care as per site specific protocols based on international guidelines (11, 13). Specifically, patients in the standard arm will receive 40–60 mL/kg of fluid bolus therapy prior to initiating inotropes, thus, 20–40 mL/kg bolus fluid *after* randomisation (in addition to the 20 mL/kg bolus fluid, or 1,000 mL fluid bolus in patients >50 kg, which has to be administered to meet enrolment criteria). Further fluid and inotrope decisions in the standard care arm are at the discretion of the treating physician. The standard care is aligned with education provided at the study sites through the Paediatric Sepsis Pathway project (Queensland Sepsis collaborative) since 2018, which advocates for the administration of sepsis resuscitation bundles as per the Surviving Sepsis Campaign.

Patients who improve in <4 h can in principle be admitted to the ward as per local practice. In patients deteriorating despite 40–60 ml/kg fluid boluses, inotropes should be commenced, and the dose rate should be increased until stabilisation occurs. Admission to PICU with appropriate monitoring should be sought as per local practice.

#### Other General Medical Care and Decisions on PICU Admission

Treating clinicians can escalate therapy as clinically indicated. Specifically, they can decide to change adrenaline dose rates, administer further fluid boluses, and consider additional inotropes or vasoactive agents to manage septic shock refractory to initial treatment. Fluid boluses in both arms can be balanced or unbalanced crystalloid (Compound Sodium Lactate, sodium chloride 0.9%, PlasmaLyte 148) or colloid (4% albumin) as per clinician preference. Other care including respiratory management, antibiotics, glucose and electrolyte control, transfusion, sedation, and extracorporeal life support should be provided at the discretion of the treating physician according to local practice. Decisions for admission to PICU must be based on medical and nursing assessment according to local practice.

### Study Outcomes

This pilot study includes feasibility outcomes, as well as clinical primary and secondary outcome measures, and proxy measures of intervention efficacy (Table 2). The *feasibility outcomes* include consent rates; compliance measures such as time to inotropes, amount of fluid delivered during the first 24 h; and protocol violations. The *clinical primary outcome* is survival free of organ dysfunction, censored at 28 days. Organ dysfunction will be assessed by pediatric Sequential Organ Failure Assessment (pSOFA) scores (23). Patients dying within 28 days of presentation will be considered as zero days to correct for the competing effect of mortality on duration of organ dysfunction. Patients discharged alive from PICU to the ward or home not requiring ongoing respiratory or renal support, and not under palliative care will be assumed to have no organ dysfunction after PICU discharge. *Secondary clinical outcomes* include PICU free survival, survival free of inotrope support and free of multi-organ dysfunction (defined as >1 organ with a pSOFA score >0), mortality, and PICU and hospital length of stay. In addition, we will obtain Pediatric Overall Performance Category and Functional Status Score at 28 days (24). Long-term follow-up will be performed at 6 months post randomisation using assessment of quality of life using neurodevelopment, quality of life and functional status, and will be reported separately. *Proxy measures of intervention efficacy* include the proportion of study participants with a blood lactate level <2 mmol/L at 6, 12 and 24 h, time to reversal of tachycardia [defined by the upper age-specific thresholds for Systemic Inflammatory Response Syndrome (22)], and time to reversal of shock (defined as cessation of inotropes for at least 4 h). Ancillary studies assessing sepsis-related costs are pre-planned but will be reported separately.

**Table 2:** Outcomes assessed.

**Outcome**	**Criterium**	**Definition**
Feasibility of the protocol	Compliance with study protocol	• Recruitment rates; proportion of eligible randomised, proportion of eligible consented using prospective consent and consent to continue. • Time to initiation of inotropes between the control and the early inotrope arm • Amount of fluid delivered (in mLs per kg) during the first 24 h between the control and the early inotrope arm • Protocol violations
Primary clinical outcome	Survival free of organ dysfunction	• Organ dysfunction defined as pediatric Sequential Organ Failure Assessment (pSOFA) score >0. Patients dying within 28 days of presentation will be allocated zero days to correct for the competing effect of mortality on duration of organ dysfunction. Patients discharged alive from PICU to the ward or home not requiring ongoing support, and not being palliative, will be assumed to have no organ dysfunction after PICU discharge.
Secondary	Patient centered outcomes	• Survival free of inotrope support at 7 days • Survival free of multiorgan dysfunction at 7 days
		• 28-day mortality • Survival free of PICU censored at 28 days • PICU length of stay • Hospital length of stay • Functional Status Score and modified Pediatric Overall Performance Category at 28 days • Neurodevelopment, Quality of life and Functional status 6 months post enrolment
	Proxy measures of intervention efficacy	• Amount of fluid (mLs per kg) received during the first h, and by 4, 12, and 24 h post enrolment • Proportion with lactate <2 mmol/l at 6, 12, and 24 h post enrolment • Time to reversal of tachycardia during the first 24 h^*^. • Time to shock reversal, defined as cessation of inotropes for at least 4 h censored at 28 days

* *Goldstein et al. (22)*.

We consider a separation of ≥10 ml/kg fluid bolus administration during the first 24 h, and a difference in inotrope commencement time of ≥20 min between the two arms as an indication of feasibility of the protocol leading to a measurable and potentially clinically relevant difference in the treatment delivered.

### Adverse Events (AEs)

The population targeted by the trial will experience a substantial degree of disease severity due to septic shock. Accordingly, consistent with best practice in ICU trials (25), we will capture major pre-defined AEs such as death, cardiopulmonary arrest, ECMO and amputations in study patients even if they are part of the natural history of the primary disease process. Any AE considered to be potentially causally related to the study intervention or which is of concern in the investigator's judgement will be reported. Specific AEs related to the intervention include, limb ischemia, extravasation injury, hypertension, arrhythmia other than sinus bradycardia or tachycardia, hyperglycaemia, abdominal compartment syndrome, pulmonary oedema and confirmed hospital-acquired infection.

Screening for adverse events will occur up until day 28, or until the time of patient discharge from hospital, whichever occurs earlier. An independent *Data and Safety Monitoring Board (DSMB)* consisting of one independent statistician, ICU specialist and emergency specialist each, who have no other involvement in the study, and who are not involved in study conduct, will review the progress and safety of the trial at regular intervals. No interim analysis for efficacy are planned.

### Data Collection

Site visits for start up and ongoing education support are provided alongside a RESPOND ED specific resource booklet to assist in time-sensitive enrolment for clinicians and trial management. Demographic variables, severity at baseline, primary end points, secondary end points, proxy measures of intervention efficiency, feasibility measures, primary diagnoses, physiological parameters, diagnoses, therapeutic interventions and documentation of deaths and other serious adverse events will be prospectively recorded into a purpose-built REDCap online database (26), hosted by The University of Queensland (Table 3). All data will be collected by trained staff at each study site using a series of electronic case report forms (eCRFs) developed by the coordinating centre. Randomized patients will be followed up by study nurses until death or 28 days post-randomisation whichever occurs first, with a follow-up review performed 6 months after randomisation. Day-28 and 6-month follow-ups will occur by phone unless the patient is still in hospital. Details on long-term follow up will be published separately. Data collection will consider the following as inotropes or vasopressors: adrenaline, noradrenaline, vasopressin, milrinone, dopamine, dobutamine. The study protocol mandates the use of adrenaline as first line inotrope.

**Table 3:** Study schedules to be captured in the study database.

**Study phase**	**Item**
Enrolment	• Screening & eligibility checks • Consent information • Baseline data including patient characteristics, treatment received, severity and physiology • Randomization
Interventions	• Time of and volume of fluid administered during first 24 h • Time of, type, and dose of inotropes administered
Early assessment	• Daily assessment of organ dysfunction using pediatric Sequential Organ Failure Assessment (pSOFA) • Organ support (respiratory and/or cardiovascular including extracorporeal membrane oxygenation, and renal support) • Laboratory markers of organ function and infection/inflammation
Late assessment	• Survival assessment at 28 days • Questionnaires by proxy on Functional Status Score and Pediatric Overall Performance Category at 28 days • Questionnaires by proxy on neurodevelopment, quality of life and functional status 6 months post enrolment

Physiological parameters and study treatment (fluids and inotropes) will be collected upon randomisation, then at 20-min intervals during the first 4 h, and at six, 12 and 24 h subsequently. The total fluid given will be captured at one, four, 12 and 24 h. Organ dysfunction and organ support will be collected upon randomisation, by 24 h and thereafter daily whilst treated in PICU for a maximum of 28 days. In addition, we will capture initiation of antibiotic therapy, intravenous steroids, duration of inotrope and/or vasopressors and respiratory support, PICU discharge, hospital discharge, death and cause of death. Protocol deviations and reasons for such will be recorded.

### Biobanking

If parents/caregivers provide consent, samples containing 1–2 mL of EDTA blood, 2.5 mL of PAXgene blood RNA, and 1–2 mL of serum will obtained at enrolment for future studies of markers of sepsis, sepsis susceptibility, and sepsis severity. Biobanking does not affect the study conduct nor randomisation. The samples will be processed, stored and assessed in batch according to standard operating procedures.

### Data Quality and Monitoring

Extensive study education is being provided to ensure medical and nursing clinicians at participating sites are well-informed about the trial. In addition, a contact phone number of study staff being available 24/7 is provided with the study booklet, flyers, and posters at the sites to enable rapid contact with research staff once a child is considered for the study. The site principal investigator will be responsible for local oversight of the study including safety reporting, ensuring that the study is conducted according to the protocol, and ensuring data integrity. Monitoring will be performed in 100% of randomized and consented patients using primary source data verification to verify randomisation allocation and commencement of intervention and consent, treatment of intravenous bolus and inotropes during the first 24 h of enrolment, organ support intervention within 28 days, PICU admission and discharge time, survival status at ED, PICU and hospital discharge, reported protocol deviations and adverse events. In 10% of randomly selected patients, we will monitor inclusion and exclusion criteria for ineligible patients, and for randomized and consented patients, baseline data, first 24 h data, demographic data, daily organ dysfunction data, ED and hospital discharge data to ensure fidelity and accuracy of obtained data. A site initiation teleconference will be conducted before site activation to ensure consistency in procedures, followed by regular videoconferences between research staff at the study sites. A data dictionary and study booklet are provided prior to study sites going live.

### Statistical Analysis Plan

#### Sample Size

A recruitment period of 24 months is the projected time to achieve the required sample size of 40 participants. The size of the pilot (*N* = 40) was chosen to reflect about 10% of a full trial to yield sufficient data in relation to feasibility. We estimate that ~50 children each year are treated for septic shock in the main participating ED. Assuming that 30–40% of children will meet exclusion criteria, and anticipating 50% enrolment rates, completion within 24 months is projected to be feasible.

#### Analysis

Using the Consolidated Standards of Reporting Trials (CONSORT) flow chart (Figure 1) (20), we will describe the number of screened patients, those meeting inclusion and exclusion criteria, the number consented, and numbers of those who withdrew consent. In addition, we will provide in the text details of the consent process, including proportion of children with prospective consent vs. consent to continue, reasons why consent to continue was used, and time from enrolment to obtaining written consent in hours (IQR). Time from randomisation to initiation of allocated treatment will be reported. Descriptive statistics will be utilised to report on demographics, clinical history and baseline clinical characteristics of patient allocated to each of the study arms (Supplementary Table 1). Statistical comparison between the characteristics of the two arms at baseline will not be undertaken. The feasibility and clinical outcome measures (Supplementary Tables 2, 3) will be compared with the estimate of the difference and corresponding 95% confidence intervals (CIs). Continuous outcomes are assumed to be non-normally distributed, and as such quantile regression will be used to generate the CIs. A test of two proportions will be used for the binary outcomes. Analyses will be by intention-to-treat. Adverse events will be presented descriptively (Supplementary Table 4).

We will graphically compare physiological measures, heart rate, systolic blood pressure, pulse pressure (systolic minus diastolic blood pressure), shock index (heart rate divided by systolic blood pressure) and use of fluid bolus volume (in ml/kg) over time, using values measured during the first 24 h, comparing the intervention group to the control group, using values measured during the first 24 h.

We anticipate that <30% of RESPOND ED study patients will be co-enrolled into a concomitant study investigating metabolic resuscitation, named RESPOND PICU (ACTRN12619000829112) (27). Given that the study is a pilot feasibility study, analyses on RESPOND ED will occur separately from RESPOND PICU.

### Current Trial Status

RESPOND ED commenced recruiting in July 2019 with a projected completion date of July 2021. Recruitment is live at the central study site Queensland Children's Hospital, and at Gold Coast University Hospital. Expansion to further centres has been delayed due to the COVID-19 pandemic.

## Discussion

Resuscitation with intravenous fluid boluses represents a key element of paediatric sepsis treatment bundles (11, 13, 28). Despite the pathophysiological rationale for liberal fluid resuscitation, there is limited evidence for this practice, and potential for harm. Subsequent to the FEAST study conducted in a low resource environment where intensive care support was not available, no similar study powered for clinical endpoints was performed in high income settings (14, 29). As a result, current pediatric Surviving Sepsis Campaign guidelines maintained the recommendation to administer a 40–60 ml/kg fluid bolus in settings where intensive care is available, based on expert opinion rather than robust evidence (11).

Different to other pilot studies on the use of fluid in critically ill children with sepsis, which primarily regulated the amount of fluid to be administered (30, 31), the study protocol stipulates the early use of adrenaline rather than prescribing how much fluid should be administered thereafter. The present pilot study was designed to capture feasibility, process of care measures, and robust severity measures. In addition, the study encompasses a comprehensive follow-up of study patients at 28-days and at 6-months to assess patient-centred outcomes such as quality of life. The pragmatic study design, embedded in an established pediatric sepsis pathway, intends to facilitate enrolment to address challenges which have been observed in other fluid trials in children with sepsis (30, 31). However, the study sample size will not permit to conduct pre-planned adjusted analyses on clinical outcomes and the study is not designed to assess the impact of co-interventions such as steroids.

In summary, this pragmatic pilot study design will provide urgently needed data to inform feasibility and design of a full trial powered to assess benefit of early inotropes in sepsis.

## Ethics Statement

The studies involving human participants were reviewed and approved by Children's Health Queensland, Brisbane, HREC/18/QCHQ/49168. Written informed consent to participate in this study was provided by the participants' legal guardian/next of kin.

## Author Contributions

LS was responsible for the initial protocol development. LS and AH were responsible for subsequent protocol refinement with input from SG, GK, MK, NP, DL, and RB at all stages of study development. AH drafted the initial manuscript. AH and LS refined and developed subsequent manuscript drafts. All authors contributed to the article and approved the submitted version.

## Group Authorship: RESPOND ED

Queensland Children's Hospital: A/Prof Luregn Schlapbach, Ms Amanda Harley, Dr Natalie Phillips, Ms Michele Cree, Dr Sainath Raman, Ms Roberta Ridolfi, Ms Natalie Sharp, Mr Angus Jones, Ms Emma Sampson.

Gold Coast University Hospital: Dr. Megan King, A/Prof Shane George, Dr. Christa Bell, Prof Gerben Keijzers, Mr Kieran Owen.

Logan Hospital: Dr Ben Lawton, Ms Vanessa Funk, Ms Brooke Lerardo.

Sunshine Coast University Hospital: Dr Paula Lister, Ms Charlotte Moore.

Royal Children's Hospital Melbourne (not recruiting, provided input into first protocol draft): Dr Elliot Long, Prof. Franz Babl.

University of Queensland, Child Health Research Centre: A/Prof Kristen Gibbons, Ms Renate Le Marsnay.

Austin Hospital and Monash University, Melbourne: Prof. Rinaldo Bellomo.

## Conflict of Interest

The authors declare that the research was conducted in the absence of any commercial or financial relationships that could be construed as a potential conflict of interest.
