# Supplementary material for: Early Resuscitation in Paediatric Sepsis Using Inotropes – A Randomised Controlled Pilot Study in the Emergency Department (RESPOND ED): Study Protocol and Analysis Plan
Source: Front Pediatr. 2021 May 31;9:663028. doi: 10.3389/fped.2021.663028 (PMC8200662; doi:10.3389/fped.2021.663028)
Supplement: Supplementary file 1 [file Data_Sheet_1.PDF]

__Supplementary Materials for:__

Early Resuscitation in Paediatric Sepsis Using Inotropes – A Pilot Randomised Controlled Pilot Study in the Emergency Department (RESPOND ED): Study Protocol and analysis plan.

Amanda Harley[1,2,3], Shane George[1,3], Megan King[3,4], Natalie Phillips[1,4], Gerben Keijzers[3,5,6], Debbie Long[1,7], Kristen Gibbons[1], Rinaldo Bellomo[9], Luregn J Schlapbach[1,10] on behalf of the RESPOND ED study group

1. Child Health Research Centre, The University of Queensland, and Paediatric Intensive Care Unit, Queensland Children`s Hospital, Brisbane, QLD, Australia
2. School of Nursing, Midwifery and Social Work, University of Queensland, QLD, Australia
3. Department of Emergency Medicine, Gold Coast University Hospital, QLD, Australia
4. Emergency Department, Queensland Children`s Hospital, QLD, Australia
5. Faculty of Health Sciences and Medicine, Bond University, QLD, Australia
6. School of Medicine, Griffith University, QLD, Australia
7. School of Nursing, Centre for Healthcare Transformation, Queensland University of Technology, Brisbane, Australia
8. Australian and New Zealand Intensive Care Research Centre, Department of Epidemiology and Preventive Medicine, Monash University, Melbourne, VIC, Australia.
9. Department of Intensive Care, Austin Hospital, Heidelberg, VIC, Australia.
10. Pediatric and Neonatal Intensive Care Unit, and Children`s Research Center, University Children's Hospital Zurich, Zurich, Switzerland

Corresponding author:
Prof. Luregn Schlapbach, MD, PhD
Head, Pediatric and Neonatal Intensive Care Unit
University Children's Hospital Zurich – Eleonore Foundation
Steinwiesstrasse 75
CH-8032 Zurich Switzerland
phone +41 44 266 71 11
email: l.schlapbach@uq.edu.au

List of supplementary Materials:

1. **Statistical analysis code.**

2. **Supplementary Mock Table 1: Baseline characteristics of infants enrolled in the RESPOND ED trial**

3. **Supplementary Mock Table 2. Feasibility outcomes per intention-to-treat analysis**

4. **Supplementary Mock Table 3. Primary and secondary clinical outcomes per intention-to-treat analysis**

5. **Supplementary Mock Table 4. Protocol violations and major adverse events**

Statistical analysis code.

The RESPOND ED Pilot study datasets is contained within a single REDCap database with records of all screened patients from the RESPOND ED and RESPOND PICU studies (data fields include date of screening, inclusion criteria, exclusion criteria, eligibility status, informed consent process, withdrawal of consent), and all consented patients (randomisation details, demographics, clinical history, baseline assessment, treatments and management, outcomes, biobanking, 6-month follow up, adverse events and protocol deviations).

The RESPOND ED study dataset will be exported from REDCap using the in-built functionality into Stata format; a Stata compatible dataset in comma-separated value (CSV) format (.csv) and Stata do-file (.do) is generated. The study dataset contains one row per screened patient per repeating event.

The code is broken into four sections, two of which are relevant for this protocol:

Part A: Transformation of primary study dataset and calculation of outcomes.

Part C: Analysis of patients enrolled in the ED study ("RESPOND ED Data Analysis.do")

The code has been uploaded to github and is available under the following link:
https://github.com/kgibbons44/RESPONDPilotAnalysis

Supplementary Mock Table 1: Baseline characteristics of infants enrolled in the RESPOND ED trial

Characteristic	Standard Care N=xx	Early Inotropes N=xx
**Age at randomisation (months)** *median (IQR)*		
**Weight (kg)** *median (IQR)*		
**Female sex** *n (%)*		
**Ethnicity**		
Caucasian *n (%)*		
Aboriginal/Torres Strait Islander *n (%)*		
Asian *n (%)*		
Maori/Pacific Islander *n (%)*		
Mixed/Other *n (%)*		
**Chronic disease** *n (%)*		
Congenital malformation *n (%)*		
Asthma *n (%)*		
Congenital heart defect *n (%)*		
Oncologic disease *n (%)*		
Cerebral palsy/severe encephalopathy *n (%)*		
Metabolic disorder *n (%)*		
Primary immunodeficiency *n (%)*		
Syndrome/genetic disorder *n (%)*		
Other *n (%)*		
**Baseline modified POPC** *median (IQR)*		
**Baseline Functional Status Score** *median (IQR)*		
**Observations at baseline**		
Heart rate *median (IQR)*		
Respiratory rate *median (IQR)*		
Systolic blood pressure *median (IQR)*		
Mean blood pressure *median (IQR)*		
Diastolic blood pressure *median (IQR)*		
Temperature *median (IQR)*		
SpO_2 *median (IQR)*		
FiO_2 at time of SpO_2 *median (IQR)*		
High-flow nasal cannulae support *n (%)*		
Non-invasive respiratory support *n (%)*		
Invasive respiratory support *n (%)*		
Capillary refill time		
Not measured *n (%)*		
<2 sec *n (%)*		
2-5 sec *n (%)*		
>5 sec *n (%)*		

**Characteristic**	**Standard Care N=xx**	**Early Inotropes N=xx**
Glasgow Coma Score *median (IQR)*		
AVPU score		
Alert *n (%)*		
Voice *n (%)*		
Pain *n (%)*		
Unresponsive *n (%)*		
**Laboratory**		
pH *median (IQR)*		
Base excess [mmol/l] *median (IQR)*		
pCO_2 [mmHg] *median (IQR)*		
Lactate [mmol/l] *median (IQR)*		
Glucose [mmol/l] *median (IQR)*		
Sodium [mmol/l] *median (IQR)*		
Chloride [mmol/l] *median (IQR)*		
Creatinine [µmol/l] *median (IQR)*		
Bilirubin [µmol/l] *median (IQR)*		
Alanine aminotransferase level [U/L] *median (IQR)*		
International Normalized Ratio *median (IQR)*		
Fibrinogen [g/L] *median (IQR)*		
Platelets [$*10^3$/µL] *median (IQR)*		
White cell count [$*10^3$/µL] *median (IQR)*		
Absolute neutrophil count [$*10^3$/µL] *median (IQR)*		
Haemoglobin [g/L] *median (IQR)*		
C-reactive protein [mg/L] *median (IQR)*		
**Organ dysfunction score**		
pSOFA *median (IQR)*		
PELOD-2 *median (IQR)*		
**Treatment**		
Total amount of fluid boluses received within the past 4 hours [ml/kg] *median (IQR)*		
Time since intravenous antibiotics were started [min] *median (IQR)*		

IQR, interquartile range; PELOD-2, Pediatric Logistic Organ Dysfunction score-2; POPC, Pediatric Overall Performance Category; pSOFA, pediatric Sequential Organ Failure Assessment

Supplementary Mock Table 2. Feasibility outcomes per intention-to-treat analysis

**Outcome**	**Standard Care N=xx**	**Early Inotropes N=xx**	**Estimate of Difference (95% CI)**
Time from screening to randomisation [min] *median (IQR)*			
Time from randomisation to commencement of intervention [min] *median (IQR)*			
Time from randomisation to adrenaline infusion commencement [min] *median (IQR)*			
Time from randomisation to inotrope infusion commencement [min] *median (IQR)*			
Inotrope infusion received within first hour of randomisation *n (%)*			
Inotrope infusion received within 24 hours of randomisation *n (%)*			
Amount of fluid received during the first 24 hours [ml/kg] *median (IQR)*			
IV Fluid Volume received during the first 1 hour [ml/kg] *median (IQR)*			
IV Fluid Volume received between >1 and 4 hours [ml/kg] *median (IQR)*			
IV Fluid Volume received between >4 and 12 hours [ml/kg] *median (IQR)*			
IV Fluid Volume received between >12 and 24 hours [ml/kg] *median (IQR)*			

CI confidence interval; IQR interquartile range; min, minutes

Supplementary Mock Table 3. Primary and secondary clinical outcomes per intention-to-treat analysis

**Outcome**	**Standard Care N=xx**	**Early Inotropes N=xx**	**Estimate of Difference (95% CI)**
**Primary clinical outcome**			
Survival free of organ dysfunction* censored at 28 days *median (IQR)*			
**Secondary clinical outcomes**			
Survival free of inotrope support at 7 days *n (%)*			
Survival free of multiorgan dysfunction** at 7 days *n (%)*			
28-day mortality *n (%)*			
Survival free of PICU censored at 28 days *median (IQR)*			
Length of stay in PICU *median (IQR)*			
Length of stay in hospital *median (IQR)*			
Modified POPC at 28 days *median (IQR)*			
Change in modified POPC from baseline *median (IQR)*			
FSS at 28 days *median (IQR)*			
Change in FSS from baseline *median (IQR)*			
**Proxy measures of intervention efficacy**			
Proportion with lactate <2mmol/l by 6 hours post randomisation n *(%)*			
Proportion with lactate <2mmol/l by 12 hours post randomisation *n (%)*			
Proportion with lactate <2mmol/l by 24 hours post randomisation *n (%)*			
Time to reversal of tachycardia censored at 24 hours [hours] *median (IQR)*			
Time to shock reversal censored at 28 days [hours] *median (IQR)*			

CI confidence interval; IQR interquartile range; PICU paediatric intensive care unit; POPC Pediatric Overall Performance Category; FSS Functional Status Score

* as measured by pediatric Sequential Organ Failure Assessment (pSOFA) score

**multi-organ dysfunction is defined as >1 organ with a pSOFA subscore of >0

Supplementary Mock Table 4. Protocol violations and major adverse events

**Variable**	**Standard Care N=xx**	**Early Inotropes N=xx**
**Protocol violations**	N=xx	N=xx
Any protocol deviation *n (%)*		
Consultant or investigator initiated withdrawal from the study prior to the finalisation of informed consent *n (%)*		
Time taken to obtain written informed consent exceeded 72 hours *n (%)*		
Written informed consent obtained but no study data collected *n (%)*		
Patient randomised but did not meet study specified inclusion/exclusion criteria for enrolment in the study *n (%)*		
Patient randomised but did not receive/commence on any study treatment(s) *n (%)*		
First treatment received/commenced is not the same as the randomised allocation *n (%)*		
Study treatment not delivered according to protocol *n (%)*		
Other		
**Major adverse events *N***	N=xx	N=xx
Any adverse event *n (%)*		
Death *n (%)*		
Cardiopulmonary arrest *n (%)*		
Extracorporeal membrane oxygenation *n (%)*		
Amputation *n (%)*		
Limb ischemia *n (%)*		
Extravasation injury *n (%)*		
Hypertension *n (%)*		
Arrhythmia *n (%)*		
Hyperglycaemia *n (%)*		
Abdominal compartment syndrome *n (%)*		

**Variable**	**Standard Care N=xx**	**Early Inotropes N=xx**
Pulmonary oedema *n (%)*		
Confirmed hospital-acquired infection *n (%)*		
Other *n (%)*		
Relatedness of the AE with the study intervention		
Not related *n (%)*		
Unlikely *n (%)*		
Possibly *n (%)*		
Probably *n (%)*		
Definitely *n (%)*		
